# Pinocembrin Inhibits the Proliferation and Metastasis of Breast Cancer *via* Suppression of the PI3K/AKT Signaling Pathway

**DOI:** 10.3389/fonc.2021.661184

**Published:** 2021-07-16

**Authors:** Xinbing Zhu, Rongnian Li, Chen Wang, Shuo Zhou, Yujia Fan, Shuang Ma, Didi Gao, Nian Gai, Jing Yang

**Affiliations:** ^1^ Department of Breast Surgery, The First Affiliated Hospital of Jinzhou Medical University, Jinzhou, China; ^2^ Department of General Surgery, Panjin Liaohe Oilfield Gem Flower Hospital, Panjin, China; ^3^ Department of Otolaryngology Head and Neck Surgery, Shengjing Hospital of China Medical University, Shenyang City, China

**Keywords:** pinocembrin, breast cancer, cell cycle, apoptosis, PI3K/AKT signaling pathway

## Abstract

The survival rate of breast cancer (BC) patients remains poor, thus the identification of safe and effective new drugs is crucial to improve therapeutic outcomes and overall survival. Pinocembrin (PCB), a pharmacologically active ingredient of Pinus heartwood, Eucalyptus, Euphorbia, Populus, and Sparattosperma leucanthum, has been widely applied for the treatment of various diseases and possesses anticancer activities. In vitro assays were performed to investigate the antiproliferation and antimetastasis activities of PCB in BC cells. A tumorigenesis assay with the use of murine BC models was performed to assess the antiproliferation activities of PCB *in vivo*. Moreover, the molecular mechanisms underlying the anticancer activities of PCB in BC cells were explored. The results showed that the anti-inhibitory and antiproliferation activities of PCB in BC might involve cell cycle (G2/M phase) arrest and apoptosis. PCB downregulated the expression levels of proteins involved in cell cycle progression and apoptosis, including cyclinB1, Cdc2, PARP1, Bcl-2, and survivin, and upregulated protein levels of cleaved PARP1, cleaved caspase3, cleaved caspase9, and BAX. In a murine subcutaneous tumor model, PCB suppressed the growth of MCF-7 cells *in vivo*. Low concentrations of PCB also significantly inhibited the migration and invasion abilities of BC cells. Mechanistically, PCB administration was correlated to suppression of the PI3K/AKT signaling pathway. Inhibition of the proliferation of BC cells by PCB involved cell cycle (G2/M phase) arrest and apoptosis *in vitro* and *in vivo*. Low concentrations of PCB also significantly inhibited the migration and invasion abilities of BC cells. These findings suggest that PCB might be an effective agent for treatment of BC patients.

## Highlights

1. Pinocembrin (PCB) inhibited the proliferation of breast cancer cells *in vitro* and *in vivo*.
2. PCB at non-cytotoxic concentrations inhibited the migration and invasion abilities of breast cancer cells.
3. PCB treatment inhibited activity of the PI3K/AKT signaling pathway through up-regulation the expression of PTEN in breast cancer cells.

## Introduction

Breast cancer (BC) accounts for about 30% of female cancers and is the second most common cause of cancer-related death among women worldwide (1, 2). BC treatment strategies including surgery, chemotherapy, radiotherapy, hormone therapy, and biological targeted therapies (3). However, due to tumor heterogeneity and multidrug resistance, the overall survival of BC patients remains less than optimal (4, 5), Thus exploring safe and effective drugs is crucial to improve the therapeutic outcomes and overall survival of BC patients.

Pinocembrin (PCB; 5,7-dihydroxyflavanone) is a pharmacologically active ingredient of Pinus heartwood, Populus, Sparattosperma leucanthum, Eucalyptus, and Euphorbia with diverse pharmacological effects (6) that has been extensively applied for the treatment of microbial infection (7), inflammation (8), ischemia-reperfusion injury (9), and atherosclerosis (10). In addition, numerous recent studies have reported that PCB has anticancer activities by targeting the cell cycle, apoptosis, and the metastatic potential of various solid tumors (11–14). However, the effect of PCB for the treatment of BC remains unclear.

Therefore, the aim of the present study was to explore the antitumor activities of PCB in BC cells and to reveal possible underlying mechanisms. The results showed that PCB had antiproliferative and antimetastatic effects *in vitro*, as well as inhibited tumorigenesis in mouse model of BC. Moreover, PCB may be function as anticancer agent *via* regulation of the PI3K/AKT pathway in BC cells.

## Materials and Methods

### Reagents and Kit

PCB was obtained from the National Institutes for Food and Drug Control (Beijing, China). Antibodies (Abs) against Cdc2 (catalog no. 10762-1-AP), cyclinA2 (18202-1-AP), cyclinB1 (55004-1-AP), cyclinD1 (26939-1-AP), cyclinE1 (11554-1-AP), Bcl-2 (12789-1-AP), BAX (50599-2-Ig), PARP1 (66520-1-Ig), survivin (10508-1-AP), PI3K (20584-1-AP), PTEN (22034-1-AP), caspase9 (10380-1-AP), and GAPDH (60004-1-Ig) were obtained from Proteintech Group, Inc. (Rosemont, IL, USA), while caspase3 (#14220), cleaved caspase3 (#9664), cleaved caspase9 (#9509), phosphorylated AKT ser473 (#4060), total AKT (#4691), LC3B (#3868), and P62 (#16177) were acquired from Cell Signaling Technology, Inc. (Danvers, MA, USA). A Cell Counting Kit-8 (CCK-8; CK04) was obtained from Dojindo Laboratories Co., Ltd. (Kumamoto, Japan). Cell cycle and cell apoptosis detection kits were purchased from Nanjing KeyGen Biotech Co., Ltd. (Nanjing, China).

### Cell Culture

Normal immortalized breast epithelial MCF-10A cells and the BC cell lines MCF-7, SKBR3, and MDA-MB-231 were obtained from the Cell Bank of the Chinese Academy of Sciences (Shanghai, China) and maintained in Dulbecco’s minimum essential medium (Gibco, Carlsbad, CA, USA) supplemented with 10% fetal bovine serum. The complexity of genetic alteration in MCF-7, MDA-MB-231, and SKBR3 cells has been shown (**Table S1**).

### CCK-8 Assay

The CCK-8 assay was used to examine the antiproliferation effects of PCB in immortalized breast epithelial cells and BC cells. Briefly, MCF-7, SKBR3, and MDA-MB-231 cells, and immortalized epithelial MCF-10A cells were seeded in the wells of 96-well plates at 2 × 104 cells/well and treated with 0, 20, 40, 80, 120, 160, 200, or 240 µM PCB for 48 or 72 h. Control cells were treated with an equal volume of vehicle (dimethyl sulfoxide). After PCB treatment for 48 or 72 h, 10 μL of CCK-8 solution was added to the wells and the plates were incubated for an additional 3 h at 37°C. Afterward, the optical density at 450 nm (OD 450) of the wells was measured with a microplate reader.

### Colony Formation Assay

Following treatment with 0, 80, 160, or 240 µM PCB for 72 h, the MCF-7 and MDA-MB-231 cells were subcultured for 2 weeks. Then, the surviving colonies were washed twice, fixed, stained with 0.5% crystal violet, photographed, and counted.

### Cell Cycle Analysis

The proportions of MCF-7 and MDA-MB-231 cells at each phase of the cell cycle were determined by flow cytometry. Briefly, the cells were seeded and cultured overnight at 37°C, and then treated with 0, 80, 160, or 240 µM PCB for 72 h. Afterward, viable MCF-7 and MDA-MB-231 cells were collected, fixed with 70% ethanol at 4°C overnight, then washed twice and treated with RNase A and propidium iodide in the dark at room temperature for 30 min. The DNA contents of the different treatment groups were determined using a flow cytometer.

### Apoptosis Analysis

The proportions of apoptotic MCF-7 and MDA-MB-231 cells were determined using an Annexin V-fluorescein isothiocyanat apoptosis detection kit. Briefly, MCF-7 and MDA-MB-231 cells were treated with 0, 80, 160, or 240 µM PCB for 72 h and then incubated with propidium iodide and Annexin V-fluorescein isothiocyanate in the dark for 30 min. A flow cytometer was used to detect apoptotic cells.

### Transfection With Small Interfering RNA (siRNA)

siRNA-PTEN and siRNA-control were synthesized by GenePharma Co., Ltd. (Shanghai, China). MCF-7 and MDA-MB-231 cells were seeded into the wells of 6-well plates at a density of 2 × 105 cells/well in Dulbecco’s minimum essential medium and then transfected with siRNA-control or siRNA targeting PTEN using Lipofectamine 2000 reagent (Invitrogen Corporation, Carlsbad, CA, USA) in accordance with the manufacturer’s instructions. After transfection for 24 h, the medium was discarded and the cells were washed and then treated with 240 µM PCB for 72 h. Downregulation of PTEN was confirmed by western blot analysis.

The transfections were conducted using the following siRNA sequences: siRNA-control: (forward) GAT CCA CTA CCG TTG TTA TAG GTG TTC AAG AGA CAC CTA TAA CAA CGG TAG TTT TTT GGA AA/(reverse) AGC TTT TCC AAA AAA CTA CCG TTG TTA TAG GTG TCT CTT GAA CAC CTA TAA CAA CGG TAGG; and siRNA-PTEN (forward) GGC GCU AUG UGU AUU AUU AdT dT/(reverse) dTd TCC GCG AUA CAC AUA AUA AU. Afterward, the cells were harvested and assayed.

### Wound Healing Assay

The migration of BC cells treated with 0, 20, 40, or 60 µM PCB for 24 h was evaluated with the wound healing assay. In brief, when the cell density reached 95%, a wound was created and the MCF-7 and MDA-MB-231 cells were seeded and incubated in serum-free medium with 0, 20, 40, or 60 µM PCB for 24 h. Images were captured under a microscope at 100× magnification. The incision width at different time points was measured and calculated. The experiment was performed independently in triplicate.

### Transwell Migration and Invasion Assay

The migration and invasion abilities of MCF-7 and MDA-MB-231 cells treated with 0, 20, 40, or 60 µM PCB for 24 h was evaluated with the transwell assay. The filter inserts of the transwell apparatus were coated with or without Matrigel. Then, 2 × 10^5^ MCF-7 and MDA-MB-231 cells were respectively seeded into the upper chamber and treated with 0, 20, 40, or 60 µM PCB in serum-free medium. After 24 h of incubation, the cells were carefully removed from the upper chamber using a cotton swab and fixed with 70% methanol, stained with 0.5% crystal violet, counted, and the permeating cells were photographed at 200× magnification.

### Western Blot Analysis

Total protein was extracted from MCF-7 and MDA-MB-231 cells using radioimmunoprecipitation assay buffer containing protease inhibitors (Proteintech, Wuhan, China). The concentrations of the protein samples were measured using the bicinchoninic acid assay. Total protein samples were separated by electrophoresis and transferred to polyvinylidene difluoride membranes, which were blocked with 5% bovine serum albumin at room temperature for 2 h and then incubated with primary Abs at 4°C overnight. The next day, the membranes were washed twice and then incubated with the secondary Abs at room temperature for 2 h. Finally, the protein bands were detected with the ChemiDoc XRS+ System (Bio-Rad Laboratories, Hercules, CA, USA).

### Animal Studies

BALB/C nude mice (age, 5–7 weeks; body weight, 15–20 g) were obtained from Beijing Vital River Laboratory Animal Technology Co., Ltd. (Beijing China) and housed in a pathogen-free facility at the Experimental Animal Center of Dalian Medical University (Dalian, China). All animal procedures were conducted in accordance with the guidelines of the Animal Care and Use Committee of Dalian Medical University. Briefly, the flank of each nude mice was subcutaneously injected with 2 × 106 MCF-7 cells. After 7 days, the mice were randomly assigned to the PCB treatment group or the control group (6 mice/group) and orally administered either 30 mg/kg of PCB in saline solution per day (PCB group) or the same volume of saline solution (control group) for 30 days. The subcutaneous tumors were measured with a caliper once every 3 days and the tumor volumes were calculated as volume = length × width2/2.

### Immunohistochemical (IHC) Analysis

IHC analysis was performed to evaluate Ki67 expression in the subcutaneous tumors. Paraffin-embedded tumor sections were deparaffinized in xylene and then rehydrated in a descending series of ethanol. For antigen retrieval, the paraffin-embedded sections were heated in citrate buffer (pH 6.0), then blocked with 3.0% hydrogen peroxide and 10% goat serum, and incubated with rabbit anti-Ki67 Ab (dilution, 1:200; Proteintech) at 4°C overnight. After incubation with the biotinylated secondary Ab, the sections were counterstained with hematoxylin, dehydrated, mounted on glass slides, and imaged under a microscope (Microphot-FX; Nikon Corporation, Tokyo, Japan) at 200× magnification.

### Statistical Analysis

Statistical analyses were performed using GraphPad Prism software (GraphPad Software Inc., La Jolla, CA, USA). Data of at least three independent experiments are presented in bar graphs as the mean ± standard deviation (SD). A probability (p) value of < 0.05 was considered statistically significant.

## Results

### PCB Inhibited the Proliferation of BC Cells *In Vitro*

**Figure 1:**
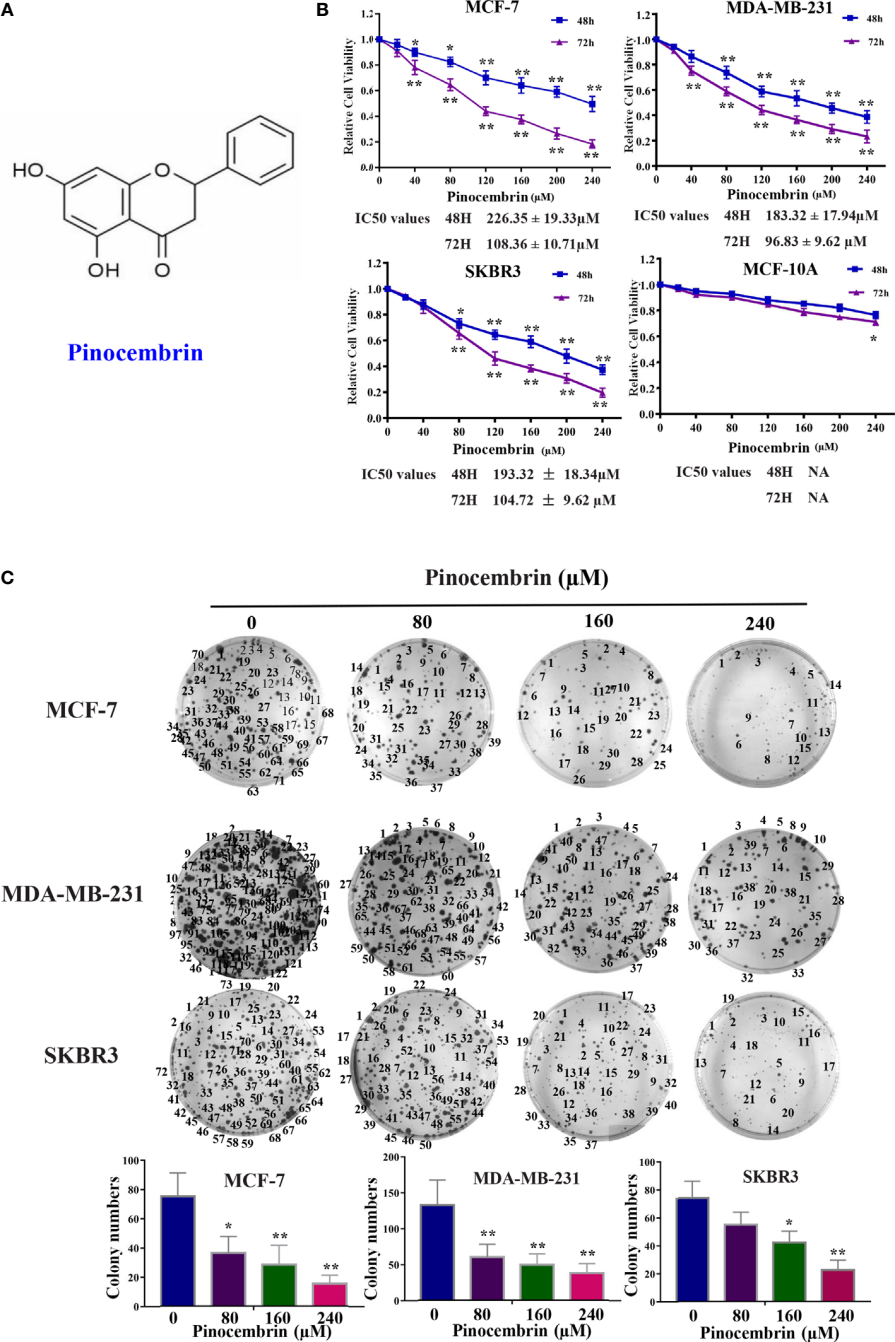
Antiproliferation effect of Pinocembrin (PCB) on breast cancer (BC) cells. **(A)** The chemical structure of PCB. **(B)** BC cells (MCF-7, MDA-MB-231, and SKBR3) and breast epithelial cells (MCF-10A) were incubated with 0, 20, 40, 80, 120, 160, 200, or 240 μM PCB for 48 or 72 h. The viabilities of cells in the different treatment groups were measured with the CCK-8 assay. **(C)** PCB treatment inhibited colony formation of MCF-7, MDA-MB-231, and SKBR3 cells. *p < 0.05, **p < 0.01 *vs*. the control group.

The chemical structure of PCB is shown in **Figure 1A**. The cytotoxic effects of PCB at 0, 20, 40, 80, 120, 160, 200, or 240 µM for 48 or 72 h were measured in normal immortalized breast epithelial cells (MCF-10A) and BC cells (MCF-7, SKBR3, and MDA-MB-231). The viability of cells treated with various concentrations PCB for 48 or 72 h was detected with the CCK-8 assay. The OD values of MCF-10A, MCF-7, SKBR3, and MDA-MB-231 cells incubated with various concentrations of PCB for 48 and 72 h are shown in **Figure 1B**. The CCK-8 assay results show that PCB inhibited the proliferation of BC cells in a concentration- and time-dependent manner. After incubation with PCB for 48 and 72 h, the half maximal inhibitory concentration values (IC50) were 226.35 ± 19.33 and 108.36 ± 10.71 µM, respectively, for MCF-7 cells, 183.32 ± 17.94 and 96.83 ± 9.62 µM for MDA-MB-231 cells, and 193.32 ± 18.34 and 104.72 ± 9.62 µM for SKBR3 cells. Notably, PCB was relatively less cytotoxic to normal immortalized breast epithelial MCF-10A cells than the three BC cell lines (**Table 1**). The colony formation assay was performed to further confirm that PCB inhibited the proliferation of BC cells. As shown in **Figure 1C**, treatment with PCB (80, 160, or 240 µM) for 72 h significantly suppressed the colony formation abilities of MCF-7, MDA-MB-231 and SKBR3 cells.

**Table 1:** The IC50 values of MCF-7, MDA-MB-231, SKBR3 and MCF-10A incubated with Pinocembrin for 48 and 72 h.

	48H	72H
MCF-7	226.35 ± 19.33μM	108.36 ± 10.71μM
MDA-MB-231	183.32 ± 17.94μM	96.83 ± 9.62μM
SKBR3	193.32 ± 18.34μM	104.72 ± 9.62μM
MCF-10A	NA	NA

### PCB Induces Cell Cycle (G2/M Phase) Arrest and Apoptosis of BC Cells

**Figure 2:**
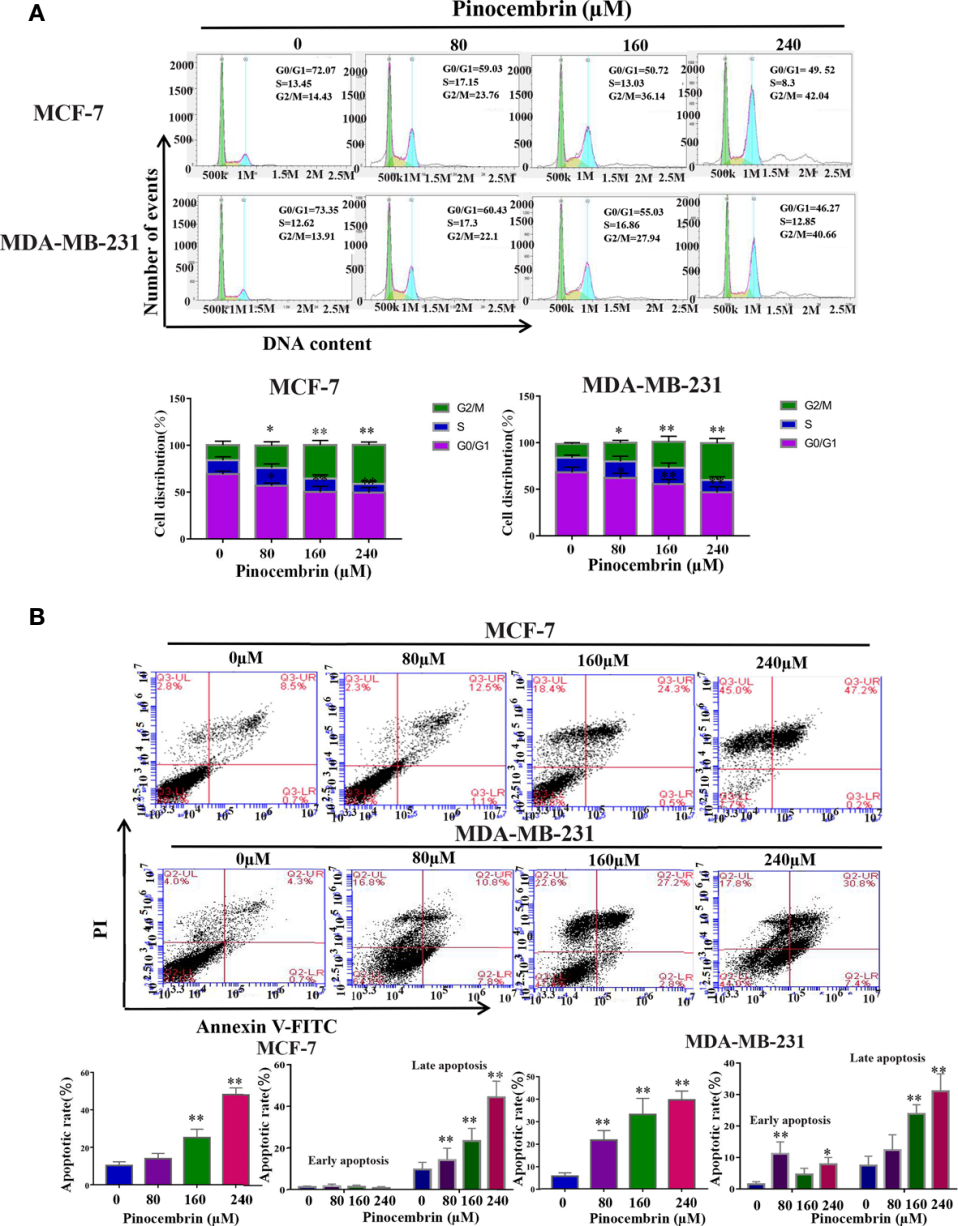
Effect of Pinocembrin (PCB) on cell cycle distribution and apoptosis in breast cancer (BC) cells. **(A)** MCF-7 and MDA-MB-231 cells were incubated with PCB (0, 80, 160, or 240 μM) for 72 h. Cell cycle distribution of MCF-7 and MDA-MB-231 cells was analyzed by flow cytometry, representative images were shown. The proportions of MCF-7 and MDA-MB-231 cells in G0/G1, S, and G2/M phases are presented in the histograms. *p < 0.05, **p < 0.01 *vs*. the control group. **(B)** MCF-7 and MDA-MB-231 cells were incubated with 0, 80, 160, or 240 μM PCB for 72 h. Annexin V/PI flow cytometry analysis was used to assess the proportions of apoptotic cells, which are presented in histograms. *p < 0.05, **p < 0.01 *vs*. the control group.

The cell cycle distribution of BC cells treated with PCB (0, 80, 160, or 240 µM) for 72 h was analyzed by flow cytometry. The proportions of MCF-7 and MDA-MB-231 cells treated with PCB at concentrations of 0, 80, 160, or 240 µM in the G2/M phase were significantly increased. The proportion of MCF-7 cells in the G2/M phase increased from 14.43% ± 1.34% at 0 µM PCB to 23.76% ± 1.87% at 80 µM, 36.14% ± 2.86% at 160 µM, and 42.04% ± 3.98% at 240 µM PCB. The proportion of MDA-MB-231 cells in the G2/M phase increased from 13.91% ± 1.23% at 0 µM PCB to 22.1% ± 2.04% at 80 µM, 27.94% ± 2.08% at 160 µM, and 40.66% ± 3.96% at 240 µM PCB. Meanwhile, the proportions of cells in the G0/G1 phase were decreased (**Figure 2A**). To verify the anti-proliferation effect of PCB, the proportions of apoptotic MCF-7 and MDA-MB-231 cells treated with PCB (0, 80, 160, or 240 μM) for 72 h were determined with the Annexin V/PI assay. The results showed that PCB treatment dramatically increased the proportions of apoptotic MCF-7 and MDA-MB-231 cells (control, 9.2% ± 0.87% and 5.0% ± 0.32%; 80 µM PCB, 13.6% ± 1.28% and 18.6% ± 1.53%; 160 µM PCB, 24.8% ± 4.27% and 30.0% ± 2.13%; 240 µM PCB, 47.4% ± 4.38% and 38.2% ± 3.26%, respectively; **Figure 2B**), suggesting that the anti-proliferation activity of PCB might be associated with G2/M phase arrest and apoptosis of BC cells.

### Effects of PCB on the Expression Patterns of Proteins Related to the Cell Cycle and Apoptosis of BC Cells

**Figure 3:**
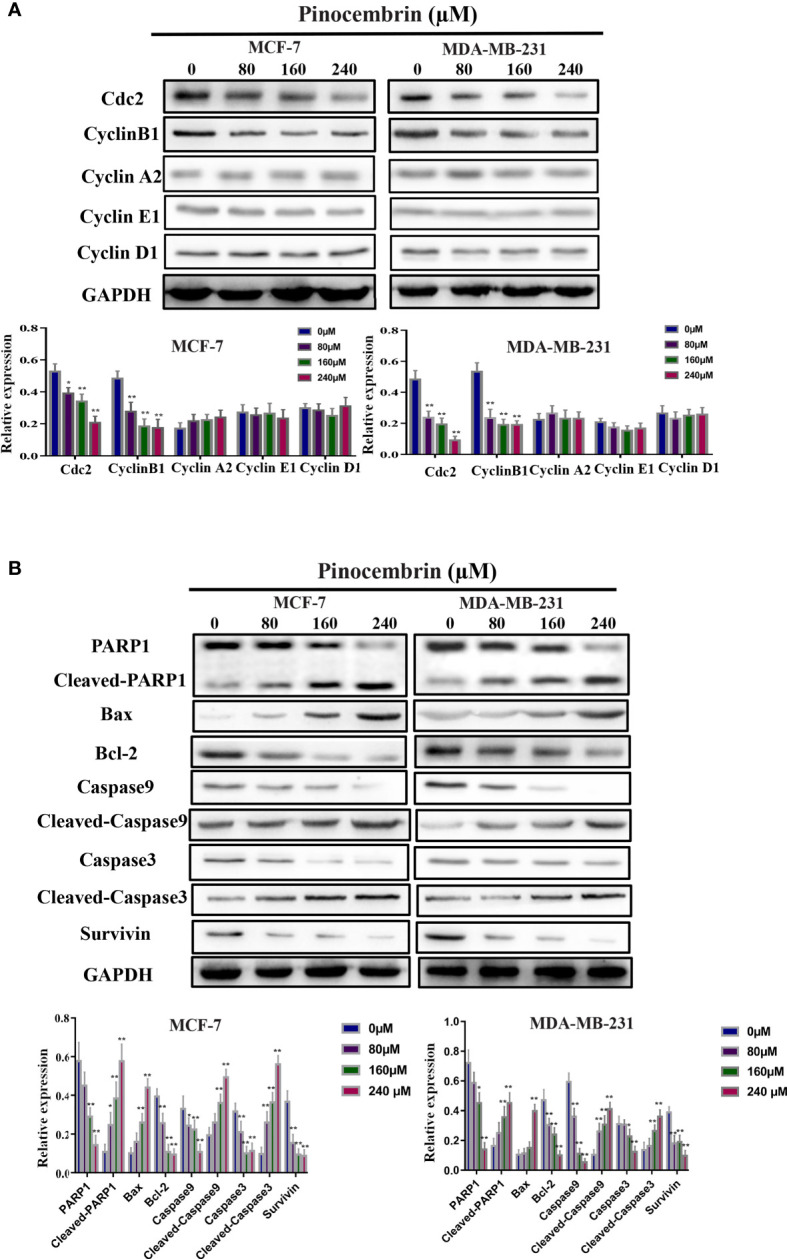
Effects of Pinocembrin (PCB) on proteins involved in the cell cycle and apoptosis of breast cancer cells. MCF-7 and MDA-MB-231 cells were incubated with 0, 80, 160, or 240 μM PCB for 72 h. **(A)** Expression of cyclinB1, Cdc2, cyclinA2, cyclinE1, and cyclinD1 were examined after PCB treatment for 72 h. **(B)** Expression of Bax, Bcl-2, PARP1, cleaved PARP1, caspase 3, cleaved caspase 3, caspase 9, cleaved caspase 9, and survivin were examined after PCB treatment for 72 h. GAPDH served as an internal control. Bands were quantified using Image J software. Each bar represents the mean ± SD of three independent experiments. *p < 0.05, **p < 0.01 *vs*. the control group.

CyclinB1 and Cdc2 play important roles in the switch from the G2 to M phase (15, 16). To explore the possible molecular mechanisms underlying G2/M phase arrest in response to PCB treatment, the protein expression levels of Cdc2, cyclinB1, cyclinA2, cyclinE1, and cyclinD1 in BC cells were measured. The results revealed that the Cdc2 and cyclinB1 protein expression levels of MCF-7 and MDA-MB-231 were significantly decreased after treatment with 80, 160, or 240 µM PCB (**Figure 3A**). PCB treatment also increased the proportion of apoptotic BC cells. Bax and Bcl-2 are known or well established to be involved in the apoptotic process (17). So, the BAX and Bcl-2 expression levels were assessed in MCF-7 and MDA-MB-231 cells after PCB treatment. The results showed that PCB treatment upregulated BAX and downregulated Bcl-2 in MCF-7 and MDA-MB-231 cells. In regard to other apoptosis-related proteins cleaved PARP1, cleaved caspase 3/9 were upregulated in MCF-7 and MDA-MB-231 cells in response to PCB treatment, while PARP1, caspase 3/9, and survivin were downregulated (**Figure 3B**). Collectively, these results showed that PCB regulated the expression levels of proteins related to the cell cycle and apoptosis in MCF-7 and MDA-MB-231 cells. Autophagy also plays an important role in tumorigenesis and development. In order to investigate whether autophagy is involved in the anticancer activity of PCB in breast cancer cells, we detected the expression levels of LC3B and p62 after 0, 80, 160, or 240 µM PCB treatment for 72 hours. The results showed that PCB treatment did not affect the autophagy process of breast cancer cells (**Figure S1**).

### Low Concentrations of PCB Suppressed the Migration and Invasion Abilities of BC Cells

**Figure 4:**
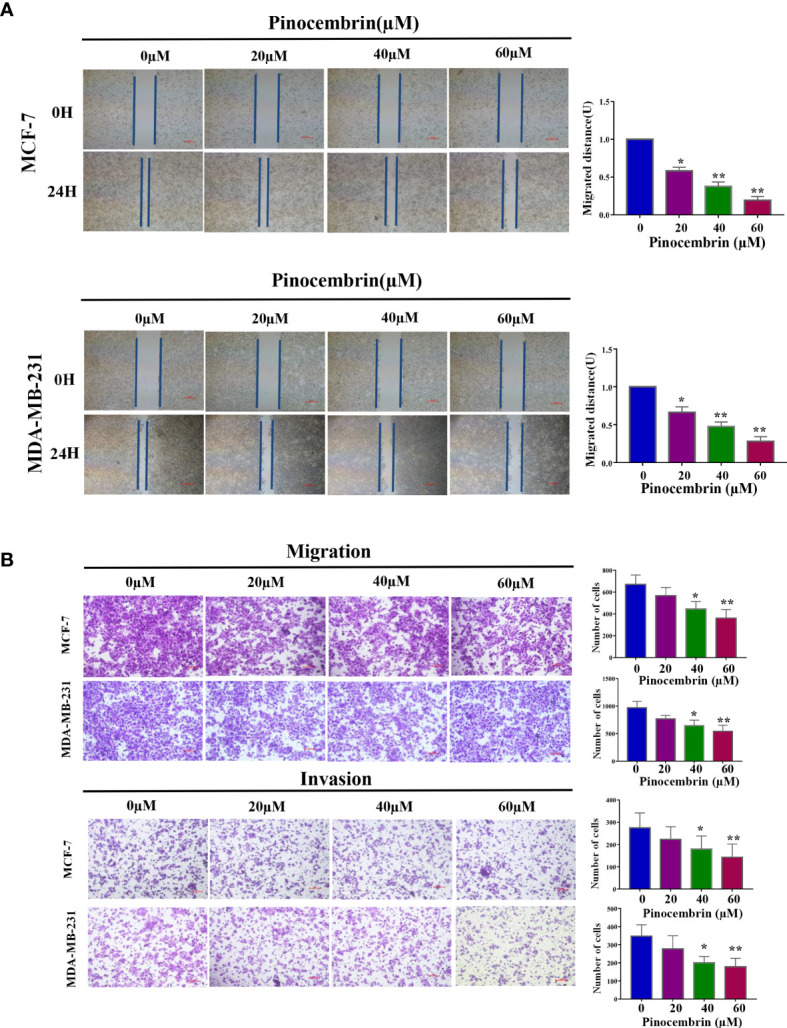
Effect of Pinocembrin (PCB) on the migration and invasion abilities of MCF-7 and MDA-MB-231 cells. **(A)** Representative images of the wound healing assay under a microscope are shown and migrated distances of MCF-7 and MDA-MB-231 cells were quantified. **(B)** The photos represent cells migrating or invading the underside of the transwell membrane. All photos were captured at 200× magnification. The cell number of each field was counted. The numbers of migrating and invading cells are shown in the histograms. *p < 0.05, **p < 0.01 *vs*. the control group.

To investigate the antimetastatic potential of PCB, the wound healing and transwell assays(with or without Matrigel) were performed to assess the effects of PCB on the migration and invasion abilities of MCF-7 and MDA-MB-231 cells. The results of the wound healing and transwell assays revealed that treatment with low concentrations (20, 40, or 60 µM) of PCB for 24 h did not induce apoptosis, but did suppress the migration and invasion abilities of BC cells in a dose-dependent manner (**Figures 4A, B**).

### PCB Suppressed Tumorigenesis of BC Cells *In Vivo*

**Figure 5:**
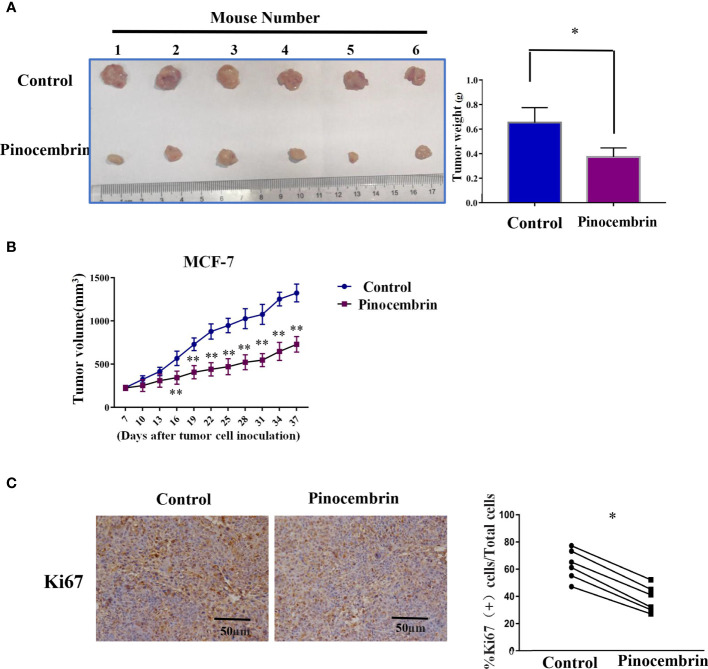
Pinocembrin (PCB) inhibits proliferation of xenografted MCF-7 cells *in vivo*. **(A)** Photograph of excised tumors from the PCB and control groups. Histogram illustrating the weight of the excised tumors. **(B)** Growth curves of subcutaneous tumors. **(C)** Ki67 expression in xenografts of the two groups by IHC analysis. *p < 0.05, **p < 0.01 *vs*. the control group.

A mouse model was used to investigate the antiproliferation activities of PCB *in vivo*. In brief, 2×106 MCF-7 cells were injected subcutaneously to the flanks of nude mice. After 7 days, the mice were randomly assigned to the PCB group (orally administered PCB at 30 mg/kg in saline solution per day for 30 days) or the control group (equal volume of saline solution per day for 30 days). As shown in **Figure 5A**, the weights of the excised tumors of the PCB-treated group were lower than those of the control group. Meanwhile, the growth curves of the excised subcutaneous tumors demonstrated that PCB treatment inhibited the growth of MCF-7 cells *in vivo* (**Figure 5B**). IHC analysis showed that KI67 expression was lower in the tumors of the PCB-treated groups than the control group (**Figure 5C**).

### PCB Treatment Inhibited Activity of the PI3K/AKT Signaling Pathway in BC Cells

**Figure 6:**
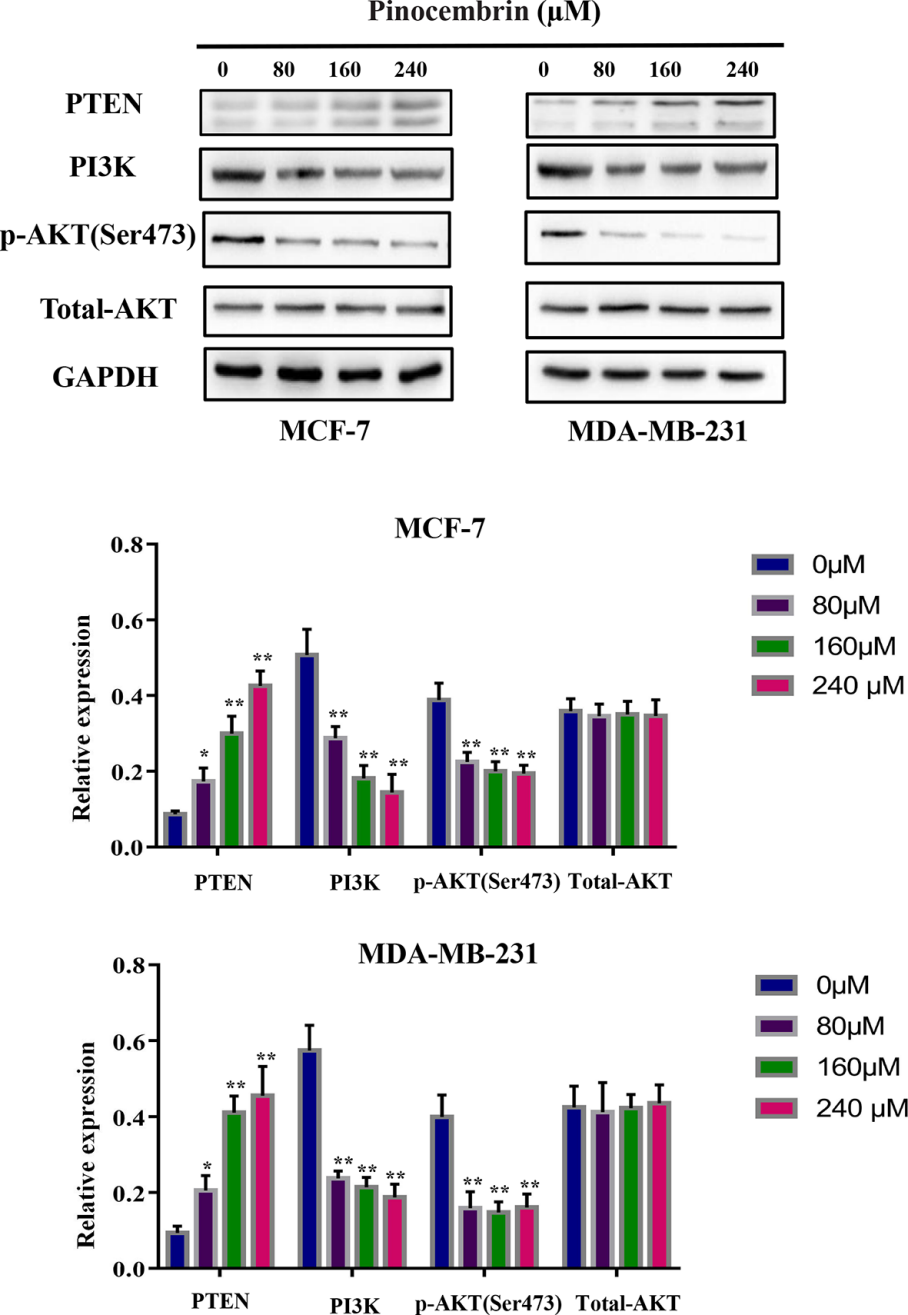
Pinocembrin (PCB) regulates the PI3K/AKT pathway. MCF-7 and MDA-MB-231 cells were incubated with PCB (0, 80, 160, or 240 µM) for 72 h. The expression levels of PTEN, PI3K, total AKT, and phosphorylated AKT were measured. GAPDH was used as an internal control. Protein bands were quantified using Image J software. Each bar represents the mean ± SD of three independent experiments. *p < 0.05, **p < 0.01 *vs*. the control group.

The PI3K/AKT pathway is involved in the proliferation, differentiation, and metastasis of various tumor cells (18). Accumulated evidence indicates that active constituents of plants used in traditional Chinese medicine, including PCB, have the ability to regulate the PI3K pathway (19). Therefore, the expression levels of PI3K, phosphorylated AKT, and total AKT were measured to investigate the activity of the PI3K/AKT signaling pathway in BC cells after PCB treatment. The results showed that PCB decreased the expression levels of PI3K and phosphorylated AKT, while total AKT protein levels remained constant. PTEN is an upstream suppressor of the PI3K/AKT signaling pathway (20). As shown in **Figure 6**, treatment with 80, 160, or 240 µM PCB for 72 h upregulated PTEN protein levels, thereby inhibiting the PI3K/AKT pathway in MCF-7 and MDA-MB-231 cells (**Figure 6**). Collectively, these findings suggest that the anticancer activities of PCB might result from inhibition of the PI3K/AKT pathway.

### Knockdown of PTEN Expression Rescued Down-Regulate the Activity of PI3K/AKT Pathway by PCB Treatment in MCF-7 and MDA-MB-231 Cells

**Figure 7:**
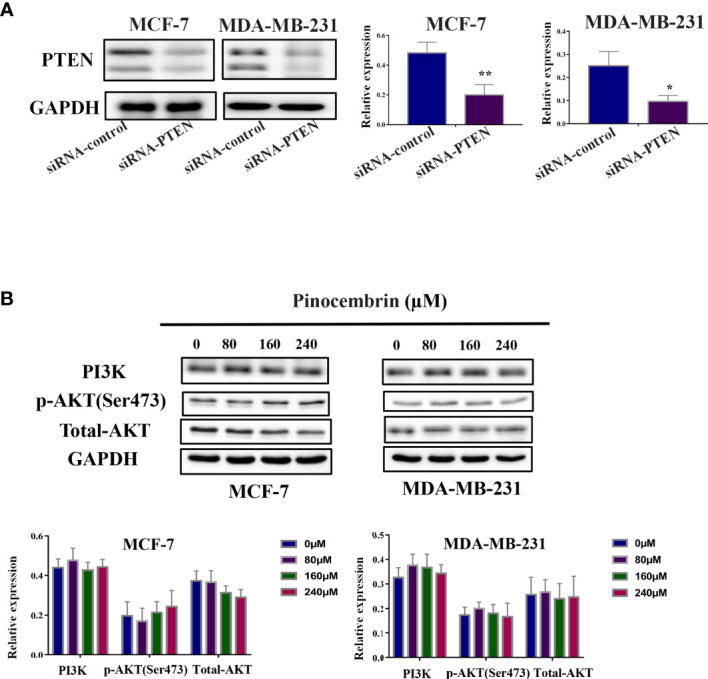
Knockdown of PTEN expression rescued down-regulate the activity of PI3K/AKT pathway by PCB treatment in MCF-7 and MDA-MB-231 cells. **(A)** Knockdown of PTEN in MCF-7 and MDA-MB-231 cells was confirmed by western blot analysis. **(B)** After PTEN protein knockdown, MCF-7 and MDA-MB-231 cells were treatment with PCB (0, 80, 160, or 240 µM) for 72 h and the expression of PI3K, total AKT, phosphorylated AKT were examined. GAPDH served as an internal control. Bands were quantified using Image J software. Each bar represents the mean ± SD of three independent experiments. *p < 0.05, **p < 0.01 *vs*. the control group.

In order to validate the role of PTEN in the anti-tumor activity of PCB,We knockdown the expression of PTEN in MCF-7 and MDA-MB-231 cells (**Figure 7A**) and investigated the protein levels of PI3K, phosphorylated AKT, and total AKT after PCB treatment. Our data showed that Knockdown of PTEN expression rescued down-regulate the activity of PI3K/AKT pathway after PCB treatment (**Figure 7B**).

## Discussion

Patients with BC still face the challenges of drug resistance and side effects of chemotherapy. Monomer active constituents of plants used in traditional Chinese medicine have potential for treatment of malignant tumors (21). Therefore, the anticancer activities of PCB in BC cells were explored as an attempt to identify effective agents with lower toxicity for treatment of BC patients. PCB, an active component extracted from Pinus heartwood, Euphorbia, Eucalyptus, Populus, and Sparattosperma leucanthum, has been extensively applied for the treatment of microbial infection (7), inflammation (8), ischemia-reperfusion injury (9), and atherosclerosis. In addition, recent studies have demonstrated the antitumor activities of PCB (11–14). Therefore, the aim of the present study was to investigate the effects of PCB on BC and to identify potential underlying molecular mechanisms.

Various monomer plant extracts have been demonstrated to inhibit the proliferation of cancer cells by inducing cell cycle arrest and/or apoptosis (22–24). Cdc2 and cyclinB1 are involved in initiating a switch from the G2 to M phase of the cell cycle (25), and some antineoplastic agents can induce G2/M arrest in cancer cells by downregulating cyclinB1 and Cdc2 protein levels (26, 27). The results of the present study showed that PCB treatment increased G2/M phase arrest *via* downregulation of cyclinB1 and Cdc2 protein levels in MCF-7 and MDA-MB-231 cells. In addition, apoptosis plays a crucial role in the regulation of cell proliferation. The induction of apoptosis of cancer cells is a common mechanism of antineoplastic agents (28). Previous studies have demonstrated that PCB inhibits the proliferation of colorectal cancer and ovarian cancer cells *via* the induction of apoptosis (11, 13, 14).

Our data also demonstrated that the proportion of apoptotic cells was increased following PCB treatment. Mitochondrial proteins reportedly directly regulate apoptosis (29). Bax and Bcl-2 have been implicated in caspase-associated apoptosis (30, 31). An increase in the Bax/Bcl-2 ratio can trigger apoptotic events, including activation of caspase3/9 and the subsequent degradation of intracellular substrates. In the present study, PCB treatment up-regulated cleaved PARP1, cleaved caspase3, and cleaved caspase9, while downregulating caspase3, caspase9, PARP1. Survivin is a member of the inhibitor of apoptosis (IAP) protein family, it inhibits apoptosis and regulates cell division (32–34). Silencing of survivin expression inhibited proliferation and induced cell cycle arrest in hela cells (35). Accordingly, our data showed PCB treatment remarkably decreased the protein lever of survivin. To date, few studies have investigated the anti-metastatic effects of PCB on tumor cells (13). To the best of our knowledge, the present study is the first to demonstrate that PCB at non-cytotoxic concentrations inhibited the migration and invasion abilities of MCF-7 and MDA-MB-231 cells.

To further investigate the possible mechanisms of the effects of PCB on cell cycle arrest, apoptosis, and cell migration/invasion, the activities of related pathways were examined. It has been reported that the PI3K/AKT signaling pathway plays important roles in the proliferation, apoptosis, and metastasis of various cancers, including BC (36, 37). Phosphorylated Akt is crucial to multiple physiological functions, such as cell proliferation, the cell cycle, apoptosis, and metastasis (38). PCB was previously reported to participate in the regulation of the PI3K/AKT signaling pathway (19). Consistent with previous studies, our data demonstrated that PCB treatment decreased the protein expression levels of phosphorylated AKT and PI3K, and increased protein expression of PTEN, while total AKT protein levels remained constant. Regulation of the PI3K/AKT signal pathway may be a potential mechanism of the anticancer effect of PCB in BC cells. In the present study, PCB downregulated the expression levels of PI3K and p-Akt, inhibited the activities of the PI3K signaling pathway, and subsequently attenuated the proliferation, migration, and metastasis abilities of MCF-7 and MDA-MB-231 cells. Meanwhile, PCB upregulated the expression of PTEN, which is upstream of the PI3K signaling pathway, similar to the common mechanism of many Chinese herbal medicines (39, 40). PTEN, as a negative upstream regulator, inhibits the activity of the PI3K signaling pathway, which may be a novel mechanism of PCB as an anticancer drug.

The anti-tumor activities of plant extracts used in traditional Chinese medicine may involve multiple mechanisms and signaling pathways, especially in different cell lines. The same drug may have different pharmacological effects on tumor cells with different backgrounds. The results of the present study suggest that the inhibitory effect of PCB on MCF-7 and MDA-MB-231 cells may be achieved by down-regulating the activities of the PI3K signaling pathway. In future studies, we plan to use whole transcriptome sequencing (RNA-Seq) and pathway analysis to assess the effects of PCB on the activities of tumor-related signaling pathways in MCF-7 and MDA-MB-231 cells in order to comprehensively and objectively elucidate the exact mechanisms underlying the anticancer activities of PCB.

The results of the present study demonstrated that PCB suppressed the growth and metastasis of BC cells both *in vitro* and *in vivo*, indicating the potential as a novel anticancer agent for the treatment of BC. PCB treatment induced cell cycle (G2M phase) arrest and apoptosis *via* regulation of the expression of proteins associated with apoptosis and the cell cycle. Also, PCB at non-cytotoxic concentrations inhibited the migration and invasion abilities of MCF-7 and MDA-MB-231 cells. Mechanistically, in response to PCB treatment, PTEN expression was upregulated, phosphorylated AKT and PI3K expression was downregulated, and the activities of the PI3K/AKT signaling pathway were suppressed. Collectively, these findings demonstrate that PCB might have beneficial applications for the treatment of BC patients.

## Data Availability Statement

The original contributions presented in the study are included in the article/**Supplementary Material**. Further inquiries can be directed to the corresponding author.

## Ethics Statement

The animal study was reviewed and approved by Animal Care and Use Committee of Dalian Medical University.

## Author Contributions

XZ, RL, JY, and CW contributed to conception and design of the study. SZ performed the statistical analysis. XZ wrote the first draft of the manuscript. YF, SM, DG, JY, and NG wrote sections of the manuscript. All authors contributed to the article and approved the submitted version.

## Funding

This present study was supported by Natural Science Foundation of Liaoning (2019-ZD-0581) and shenyang science and technology planning project (19-112-4-084).

## Conflict of Interest

The authors declare that the research was conducted in the absence of any commercial or financial relationships that could be construed as a potential conflict of interest.

## Supplementary Material

Supplementary Figure 1 The expression levels of LC3B and P62 were measured after incubated with PCB (0, 80, 160, or 240 µM) for 72 h. GAPDH was used as an internal control. Protein bands were quantified using Image J software. Each bar represents the mean ± SD of three independent experiments.

Supplementary Table 1 The complexity of genetic alteration in MCF-7, MDA-MB-231, and SKBR3 cells has been shown.
