# Supplementary figures and images for: Pinocembrin Inhibits the Proliferation and Metastasis of Breast Cancer via Suppression of the PI3K/AKT Signaling Pathway
Source: Front Oncol. 2021 Jul 16;11:661184. doi: 10.3389/fonc.2021.661184 (PMC8322951; doi:10.3389/fonc.2021.661184)

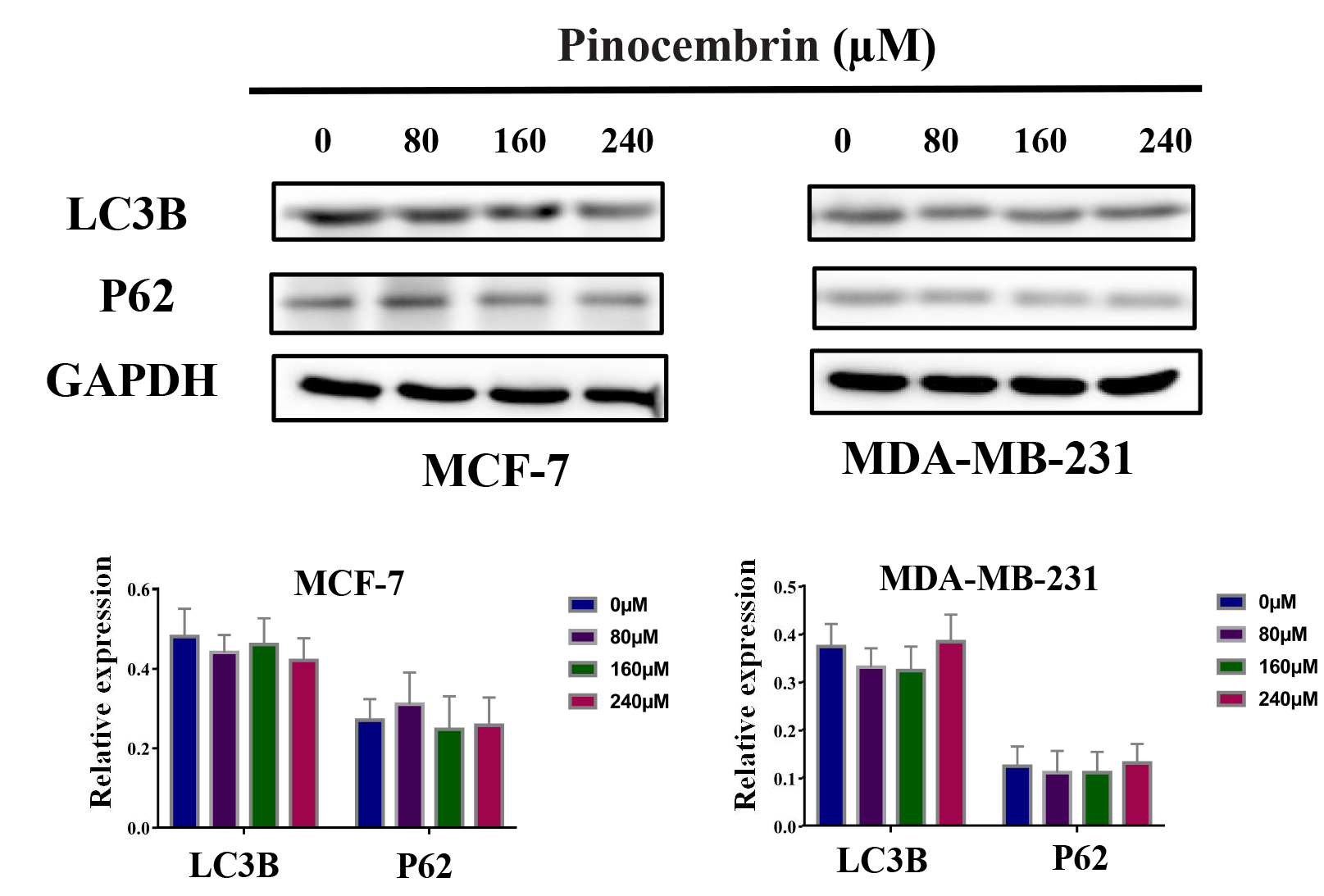

Supplement: Supplementary Figure 1 — The expression levels of LC3B and P62 were measured after incubated with PCB (0, 80, 160, or 240 µM) for 72 h. GAPDH was used as an internal control. Protein bands were quantified using Image J software. Each bar represents the mean ± SD of three independent experiments. [file Image_1.tif]
